# *Paracoccidioides brasiliensis* habitat: far beyond armadillo burrows?

**DOI:** 10.1590/0074-02760200208

**Published:** 2020-07-20

**Authors:** Priscila Marques de Macedo, Bruno de Souza Scramignon-Costa, Rodrigo Almeida-Paes, Luciana Trilles, Larissa Siston Cosendey de Oliveira, Rosely Maria Zancopé-Oliveira, Antonio Carlos Francesconi do Valle, Bodo Wanke

**Affiliations:** 1 Fundação Oswaldo Cruz-Fiocruz, Instituto Nacional de Infectologia Evandro Chagas, Rio de Janeiro, RJ, Brasil

**Keywords:** outbreak, environment, paracoccidioidomycosis, Paracoccidioides brasiliensis

## Abstract

*Paracoccidioides* spp. isolation from environmental samples is rare and hardly reproducible. Molecular techniques have facilitated the fungal detection. However, it can be still difficult. Some strategies to enhance the capacity of DNA detection have been adopted, including the analysis of soil samples belonging to the habitat of animals from which *Paracoccidioides* spp. have already been isolated, notably armadillo burrows. To date, the detection of *Paracoccidioides* spp. has not yet been reported from outbreak hotspots. Clusters and outbreaks of acute paracoccidioidomycosis (PCM), usually a more severe clinical form, have currently occurred in urban areas being associated to climate changes, deforestation, and great constructions. These occurrences potentially signalise the fungus’ environmental niche, a riddle not yet solved. The authors performed an environmental investigation in a deeply disturbed area, after a highway construction in Rio de Janeiro, Brazil, where a recent outbreak of acute PCM occurred. Specific DNA sequences of *Paracoccidioides brasiliensis* were detected in shallow soil samples around the highway, reinforcing the association between the road construction and this PCM outbreak.

Acute forms of paracoccidioidomycosis (PCM), a systemic mycosis caused by inhalation of *Paracoccidioides* spp. conidia present in soil from endemic areas, are usually more severe and less common than the chronic PCM form.^1^ In 2017 we reported an outbreak of acute PCM after a highway construction in Rio de Janeiro, Brazil, involving eight young patients and characterised by severe clinical presentations, complications, and one death.^2^ After this outbreak, 18 additional cases of acute PCM were diagnosed in the same study area, a rate 4.3 times higher than that expected for this period. Considering the outbreak, the road construction, the ongoing diagnosis of new cases of acute PCM, and the knowledge that the fungus lives in soil, the authors performed an environmental investigation along the roadside to reinforce the hypothesis that the outbreak was related to the road construction.

We collected soil samples (around 100 g each) in the surroundings of the roadside (no more than 300 m away). Nine samples were obtained nearby the epicenter of the cases’ occurrence (Nova Iguaçu municipality, 12 km away from the residence of one patient previously described), and nine soil samples at Seropédica, another municipality where a severe acute PCM case recently occurred (8 km away from the residence of this patient). Table depicts the GPS coordinates, dates, seasons, and climatic conditions of the environmental investigations. Soil samples were processed for culture and DNA extraction within 24 h after field work. For culture, 1 g of each soil sample was diluted in 9 mL sterile saline, vortexed for 10 min and heavy particles were allowed to settle down for 5 min. Serial tenfold dilutions of the upper homogenous suspensions were plated on Mycosel Agar (Difco, Sparks, MD, USA) at 25ºC for 60 days. DNA extraction was conducted using the DNeasy® PowerSoil® Kit (Qiagen, Hilden, Germany). Molecular analyses through nested-polymerase chain reaction (PCR) were performed as described.^3^ The following controls were used: (1) soil artificially seeded with *Paracoccidioides brasiliensis* (positive control), following the protocols published by Theodoro et al.;^3^ (2) *P. brasiliensis* Pb18 strain (another positive control); (3) beach sand from Barra da Tijuca, Rio de Janeiro (negative control); (4) all PCR reagents without any DNA template (internal negative control). Cultures did not yield colonies of *P. brasiliensis*, however, the presence of specific DNA sequences of *P. brasiliensis* was detected in both sites studied: two soil samples from Seropédica, and one soil sample from Nova Iguaçu (Figure). DNA bands from the nested-PCR were excised from the gel, purified with the illustra^TM^ GFX^TM^ PCR DNA and Gel Band Purification Kit (GE Healthcare, Buckinghamshire, UK), sequenced at the sequencing platform at Oswaldo Cruz Foundation - PDTIS/Fiocruz, and *P. brasiliensis* was revealed as best hit in all three positive samples after BLAST search (sequence numbers MT726207 to MT726209).

**TABLE GPS:** coordinates, dates, seasons, and climatic conditions of the environmental investigations

Place	GPS	Date	Season	Temperature	Last rainfall	
					Date	Precipitation
Seropédica	S 22º46’52.3’’ W 043º45’27.1’’	07/12/2019	Winter	26ºC - 14ºC	8 days before	2.0 mm
Nova Iguaçu	S 22º40’37.3’’ W 043º24’17.7’’	03/16/2020	Summer	34ºC - 24ºC	12 days before	2.0 mm

**Figure:**
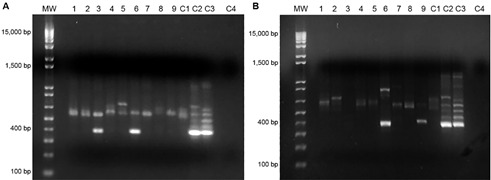
Nested polymerase chain reaction (PCR) results, with specific *Paracoccidioides brasiliensis* ITS primers, of DNA extracted from soil samples of: (A) Seropédica, and (B) Nova Iguaçu municipalities. MW: 1 kb plus DNA Ladder (Invitrogen); Lanes 1 to 9: soil samples from each site; C1: negative soil control (beach sand); C2: positive soil control; C3: *Pb*18 strain; C4: negative internal control.

*Paracoccidioides* spp. isolation from soil samples is a great challenge. This pathogen is fastidious in culture and lives in tropical areas where the high fungal diversity and concentration in soil may inhibit its growth and isolation. Some other hypotheses raised in the literature consider a more selective microniche, a transitory and short saprobic phase, or a possible obligatory parasite phase in wild animals supported by the high frequency of infected armadillos in some endemic areas.^4^ Molecular techniques have facilitated the detection of *Paracoccidioides* spp. in environmental samples. However, this detection can be still difficult as it was postulated a low sporulation capacity and a very low inoculum level of this fungal agent in soil.^4^ ^,^ ^5^ In this context, some strategies to enhance the capacity of DNA detection have been adopted: the DNA extraction in triplicate from each soil sample, including a final step of concentration; the nested PCR due to its higher capacity of DNA amplification; and the analysis of soil samples collected from the habitat of animals from which *Paracoccidioides* spp. have already been isolated.^6^ Our work was developed in a deeply disturbed area, the samples were collected in the roadside, 10 cm of soil depth, and DNA extraction was performed once from each sample. The two sites where samples were collected had positive results, suggesting that the environmental disruption related to the road construction was responsible for a greater exposure of infective *Paracoccidioides* spp. propagules and, consequently, to the reported PCM outbreak.

The One Health concept (WHO, 2017) recognises the connection between people, animals, and the environment, encouraging scientists to investigate human’s role in the emergency of diseases due to an imbalance in this connection.^7^ To date, reports warn about alterations in PCM epidemiology related to climate and rainfall pattern changes, as well as other environmental human disruptions such as deforestation and big constructions, causing more severe PCM clinical profiles in urban areas.^2^ ^,^ ^8^ ^,^ ^9^ Traditionally, PCM is characterised by a long period of latency, absence of reported outbreaks, and paucity of acute cases as well as confirmed subclinical infections, which historically tended to hinder determination of the possible sources of infection.^10^

The findings herein reported reinforce our original hypothesis of association between the road construction and the PCM outbreak in Rio de Janeiro, Brazil, opening up new perspectives to other possible environmental sources of PCM infection.
